# A Comparative Analysis of Gene Expression Profiles during Skin Regeneration in *Mus* and *Acomys*

**DOI:** 10.1371/journal.pone.0142931

**Published:** 2015-11-25

**Authors:** Jason Orr Brant, Maria-Cecilia Lopez, Henry V. Baker, W. Brad Barbazuk, Malcolm Maden

**Affiliations:** 1 Department of Biology, University of Florida, Gainesville, Florida, United States of America; 2 UF Genetics Institute, University of Florida, Gainesville, Florida, United States of America; 3 Department of Molecular Genetics and Microbiology, University of Florida, Gainesville, Florida, United States of America; Medical College of Georgia, UNITED STATES

## Abstract

The African spiny mouse (*Acomys* spp.) can heal full thickness excisional skin wounds in a scar-free manner with regeneration of all dermal components including hair and associated structures. Comparing *Acomys* scar-free healing from *Mus* scarring identifies gene expression differences that discriminate these processes. We have performed an extensive comparison of gene expression profiles in response to 8mm full-thickness excisional wounds at days 3, 5, 7 and 14 post-wounding between *Acomys* and *Mus* to characterize differences in wound healing, and identify mechanisms involved in scar-free healing. We also identify similarities with scar-free healing observed in fetal wounds. While wounding in *Mus* elicits a strong inflammatory response, wounding in *Acomys* produces a moderated immune response and little to no increase in expression for most cytokines and chemokines assayed. We also identified differences in the ECM profiles of the *Acomys* wounds, which appear to have a collagen profile more similar to fetal wounds, with larger increases in expression of collagen types III and V. In contrast, *Mus* wounds have very high levels of collagen XII. This data suggests that an overall lack of induction of cytokines and chemokines, coupled with an ECM profile more similar to fetal wounds, may underlie scar-free wound healing in *Acomys* skin. These data identify candidate genes for further testing in order to elucidate the causal mechanisms of scar-free healing.

## Introduction

Wound healing is a dynamic and highly coordinated series of complex events, that has been described extensively [1]. In order to attain tissue integrity following wounding in adult mammalian tissue, the healing process occurs in three overlapping phases: inflammation, tissue formation and tissue remodeling. Immediately following wounding, hemostasis occurs in the presence of aggregated platelets. During the inflammatory phase first neutrophils, and subsequently monocytes, infiltrate the wound and eliminate tissue debris and contaminating bacteria through phagocytosis. Granulation tissue is formed during the tissue formation phase of wound healing. This is characterized as a loose matrix of fibronectin and immature collagen fibers supporting migration of proliferative fibroblasts and vascularization of the wound bed. This newly formed tissue is then covered by a new wound epidermis formed by migration of cells from the wound edge, and results in the restoration of tissue continuity of the wound. In the final phase of wound healing the granulation tissue is remodeled, which results in an altered collagen profile and reduced vascularity. The end result of this series of events is scar tissue comprised of non-functional dermal tissue covered by a smooth, hairless epidermis.

In contrast, wounding of fetal mammalian tissue, up to the middle of the third trimester, results in scar free healing. This phenotypic difference in wound healing outcomes has lead to numerous studies comparing fetal and adult wound healing in order to determine what is responsible for the improved outcome (reviewed in [2]). These studies have been highly informative and have shown differences in several processes involved in wound healing between adult and fetal tissue; fetal wounds show a blunted inflammatory response, reduced fibrosis and vascularization, and a different extracellular matrix (ECM) profile.

A consistently observed characteristic associated with fetal skin wounding is a substantially blunted inflammatory immune response relative to that initiated from adult wounding [3–5]. This is due in part to reduced levels of *Pdgfa*, *Tgf-β*1 and *Tgf-β2* [6]. The number of immune cells present in fetal wounds is also decreased, with fetal wounds having fewer macrophages that are present for a shorter duration [7]. Fetal wounds also contain fewer neutrophils and they demonstrate reduced phagocytic activity [8, 9]. The reduced presence of immune cells results in reduced levels of inflammatory cytokines and growth factors such as Tgf-*β*1 [10].

There are also differences in the ECM composition between fetal and adult wounds. The ECM has been shown to regulate cytokines and growth factors and to promote cell migration through the wound, and as such it is considered an important component of wound healing [11]. The fetal ECM has a higher ratio of collagen III:collagen I and higher levels of collagen V as well [12–14]. In addition, fetal wounds have increased levels of matrix metalloproteinases (MMPs) and lower levels of tissue inhibitors of matrix metalloproteinases (TIMPs), with these ratios reversed in adult wounds [15–17], suggesting that there is more active degradation and remodeling of wound tissue in fetal wounds.

Despite the extensive data comparing fetal scar-free and adult scarring mechanisms, very little has successfully been translated into improved outcomes following wounding in adult tissue. This is in part due to the inherent differences between fetal and adult tissues. Furthermore, our ability to compare scar-free healing vs scarring in two adult tissues has, until recently, been limited to contrasting wound repair between evolutionarily distant vertebrates. Although Urodeles have a remarkable capacity for regeneration [18, 19], it is difficult to translate findings from such disparate groups into improved clinical outcomes.

We have recently shown that the African spiny mouse, a member of the *Muridae* approximately 20MY diverged from *Mus* [20], can heal full thickness excisional skin wounds in a scar-free manner with regeneration of all dermal components including regeneration of hair and associated structures [21]. This discovery enables comparison of scar-free healing vs scarring in two closely related adult mammalian species.

In order to examine differences in wound healing between *Acomys* and *Mus* and to identify genes driving the mechanism of scar-free healing, we have performed an extensive comparison of gene expression profiles for 8mm full-thickness excisional wounds at days 3, 5, 7 and 14 post-wounding. Additionally, we asked whether scar-free healing in *Acomys* shares any similarities with scar-free healing described above for fetal tissues. Here we present evidence of expression differences in genes participating in several aspects of wound healing between *Mus* and *Acomys*, especially in the inflammatory pathway and the digestion and deposition of the ECM. While wounding in *Mus* elicits a strong inflammatory response, the response in *Acomys* wounds is substantially muted and exhibits little to no increase in expression for most cytokines and chemokines assayed. We also show that the ECM profiles of the *Acomys* wounds indicate large increases in expression of collagen types III and V, and little collagen XII relative to *Mus* wounds, indicating they are more similar to fetal rather than adult wounds. This data suggests that an overall lack of induction of cytokines and chemokines, coupled with an ECM profile more similar to fetal wounds, may be responsible for this remarkable scar-free wound healing observed in *Acomys* skin.

## Methods

### Animals

All experiments were performed following guidelines of the *Guide for the Care and Use of Laboratory Animals of the National Institutes of Health*. The protocol was approved by the Institutional Animal Care and Use Committee (IACUC) at the University of Florida (Protocol Numbers: 201203505 (*Mus*) and 201207707 (*Acomys*)), and all animals were housed under the care of the University of Florida’s Animal Care Services. All surgeries were performed under isoflurane inhalational anesthesia and all efforts were made to minimize suffering. CD-1 outbred mice (Charles River Laboratories) were used as *Mus* controls for all experiments. Animals were between 6 months to 1 year old at time of experiments; One to two 8-mm biopsy punches were excised from the dorsal skin of anaesthetized, shaved animals. Excised skin was immediately placed in RNALater (Qiagen Cat. 76104) at room temperature for 24 hours and then stored at -80°C. Wounds were excised at appropriate time points (3, 5, 7 or 14 days post wounding), excluding surrounding normal skin, and stored as before for normal skin.

### RNA Extraction

Tissue samples were removed from -80°C and thawed at 4°C for 24 hours before processing. Total RNA was extracted using RNeasy Fibrous Tissue Mini Kit (Qiagen Cat. 74704) following the manufactures recommended protocol, with tissue homogenization being performed using a rotor stator type tissue homogenizer (ProScientific Bio-Gen PRO200 Homogenizer; Multi-Gen 7XL Generator Probes) in RLT Buffer (Qiagen Cat. 74704). RNA quality was assayed using an Agilent 2200 TapeStation (Agilent, Andover, MA). All samples had a RIN score ≥ 6.0.

### RT-qPCR analysis

cDNA was generated from 500ng of RNA using iScript^TM^ cDNA Synthesis Kit (Bio-Rad cat. no. 170–8891) following manufacturer’s protocol. Real-time PCR was performed using SsoFast^TM^ EvaGreen® Supermix (Bio-Rad cat. no. 172–5200) following manufacturer’s protocol on a Bio-Rad CFX-96 Real-Time PCR Detection System. Fold change of expression was calculated using the ΔΔCt relative expression method [22]. Change in expression was expressed as normal skin versus wounded skin. Expression values for RT-PCR arrays (RT^2^ Qiagen cat. no. PAMM-121Z) were calculated using *Hsp90ab1* (Heat shock protein 90 alpha, class B member 1) and *Gusb* (Glucuronidase, beta) as reference genes.

### Microarray analysis

100ng of total RNA was processed for microarray analysis using the GeneChip® WT PLUS Reagent Kit (Affymetrix, Santa Clara, CA) following manufacturer’s recommended protocols. cDNA (5.5ug) was fragmented, terminally labeled and hybridized to Affymetrix GeneChip® Mouse Gene 2.0 ST Array (MoGene 2.0) for 16 h at 45°C and washed following Affymetrix fluidics protocols FS450_001. Microarrays were normalized using the robust multiarray average (RMA) method as implemented in Partek Genomics Suite 6.6 (Partek Incorporated, St Louis MO). Only annotated probe sets were used in the subsequent analysis. BRB-ArrayTools (version 4.3.0 Stable Release, developed by Richard Simon & BRB-ArrayTools Development Team (http://linus.nci.nih.gov/BRB-ArrayTools.html)) was utilized to identify significant genes (p<0.001) using the class prediction tool. Leave-one-out-cross-validation studies and Monte Carlo simulations were performed using BRB-Array Tools. Venn diagrams were generated using the R package VennDiagram (http://cran.r-project.org/web/packages/VennDiagram/index.html).

Pathway analysis was performed using WEB-based Gene Set Analysis Toolkit (WebGestalt) [23, 24]. Gene Ontology enrichment analysis summarization was performed using REViGO [25]. Semantic similarity-based scatterplots were generated using REViGO and modified in R using the REViGO generated R script.

### Data availability

The microarray dataset discussed in this publication has been deposited in NCBI’s Gene Expression Omnibus and is accessible through GEO Series accession number GSE74387. (http://www.ncbi.nlm.nih.gov/geo/query/acc.cgi?acc=GSE74387)

## Results

### Pathway focused RT-PCR arrays

In order to elucidate the observed differences in wound healing between *Mus* and *Acomys* we examined the expression profile of full-thickness excisional wounds at 3 and 5 days post-wounding (compared to normal skin) using pathway-focused RT-PCR arrays with 84 genes targeted for involvement in wound healing (RT^2^ Qiagen cat. no. PAMM-121Z). In *Mus* there were 21 genes up and 1 gene downregulated at day 3 post-wounding, and 24 up and 3 downregulated genes at day 5 post wounding, as compared to normal skin. In *Acomys*, there were 14 genes up and 1 gene downregulated at day 3, and 14 up and 2 downregulated at day 5 post wounding compared to normal skin (S1 Table) (p-values ≤ 0.01). At both day 3 and 5 in the *Mus*, the top upregulated genes are those involved in the inflammatory pathway, as well as neutrophil and macrophage activation and migration, with a substantial increase in c-x-c motif chemokines, interleukins and growth factors (Table 1). In comparison, the top upregulated genes in the *Acomys*, at both days 3 and 5, are those involved in tissue remodeling, including extracellular matrix degradation and deposition, with the inflammatory response being quite blunted (Table 1).

**Table 1:** Differentially expressed genes in day 3 and 5 wounds. Differentially expressed genes in day 3 and 5 wounds, compared to normal skin, within each species. Bold entries are those with a p-value ≤0.01.

	*Mus musculus*			*Acomys cahirinus*	
Gene	Day 3 vs 0	Day 5 vs 0	Gene	Day 3 vs 0	Day 5 vs 0
Cxcl3	**47963.253**	**109235.609**	Ptgs2	**433.720**	**902.276**
Csf3	**8369.125**	**9126.401**	Csf3	**181.376**	**294.590**
Cxcl5	**4067.714**	**4464.773**	Mmp9	**90.509**	**65.085**
Il1b	**358.635**	**220.279**	Tnf	**54.254**	**31.358**
Serpine1	**233.382**	**275.948**	Actc1	**26.960**	5.313
Cxcl1	**151.834**	**239.431**	Mmp2	**7.223**	**7.881**
Ptgs2	**70.466**	**67.940**	Tgfb1	**6.862**	**8.716**
Timp1	**68.761**	**56.027**	Wisp1	6.127	9.138
Plaur	**48.848**	**62.479**	Vegfa	**5.741**	**7.545**
Tnf	**27.552**	**29.862**	Col5a3	**4.811**	6.196
Col5a3	**16.050**	**17.202**	Itgb3	**4.363**	**9.454**
Mmp9	**13.534**	**14.893**	Itgb1	**3.700**	**3.875**
Csf2	13.388	34.471	Col5a1	**3.623**	**8.626**
Wisp1	**10.296**	**15.814**	Col5a2	**3.489**	**7.405**
Ccl7	**7.142**	**11.577**	Ctsg	2.321	N/A
Il10	**6.664**	**6.198**	Il1b	2.231	17.350
Col14a1	**4.364**	**7.799**	Fgf2	2.126	**4.205**
Vegfa	**4.285**	**6.506**	Plat	1.927	**3.433**
Hgf	3.851	**5.408**	Cxcl3	1.775	3.308
Plau	3.740	**3.786**	Mif	**1.566**	1.807
Plat	**3.648**	4.283	Col4a3	1.561	-12.627
Itgb3	**3.250**	**4.290**	Col14a1	1.552	4.846
Mif	**3.030**	**3.755**	Col3a1	1.528	**3.313**
Tgfb1	**2.937**	**3.545**	Timp1	1.294	3.095
Actc1	2.408	**12.429**	Plaur	-1.337	N/A
Col5a1	2.088	2.805	Itgb6	-1.494	**-5.143**
Itgb1	2.062	2.273	Csf2	-2.768	-1.273
Fgf7	1.901	**2.918**	Il4	**-6.935**	**-5.590**
Col5a2	1.671	2.118	Ccl7	N/A	N/A
Il4	1.208	-1.557	Cxcl1	N/A	N/A
Mmp2	1.158	1.743	Cxcl5	N/A	N/A
Ifng	-1.180	**-2.665**	Fgf7	N/A	N/A
Col3a1	-2.144	-1.551	Hgf	N/A	N/A
Itgb6	**-2.272**	-1.449	Ifng	N/A	N/A
Fgf2	-2.445	-2.325	Il10	N/A	N/A
Ctsg	**-8.594**	**-2.514**	Plau	N/A	N/A
Col4a3	-10.323	**-5.921**	Serpine1	N/A	N/A

#### Examining expression differences in genes known to participate in the inflammation pathway and the extra-cellular matrix during cutaneous wound healing

**Fig 1:**
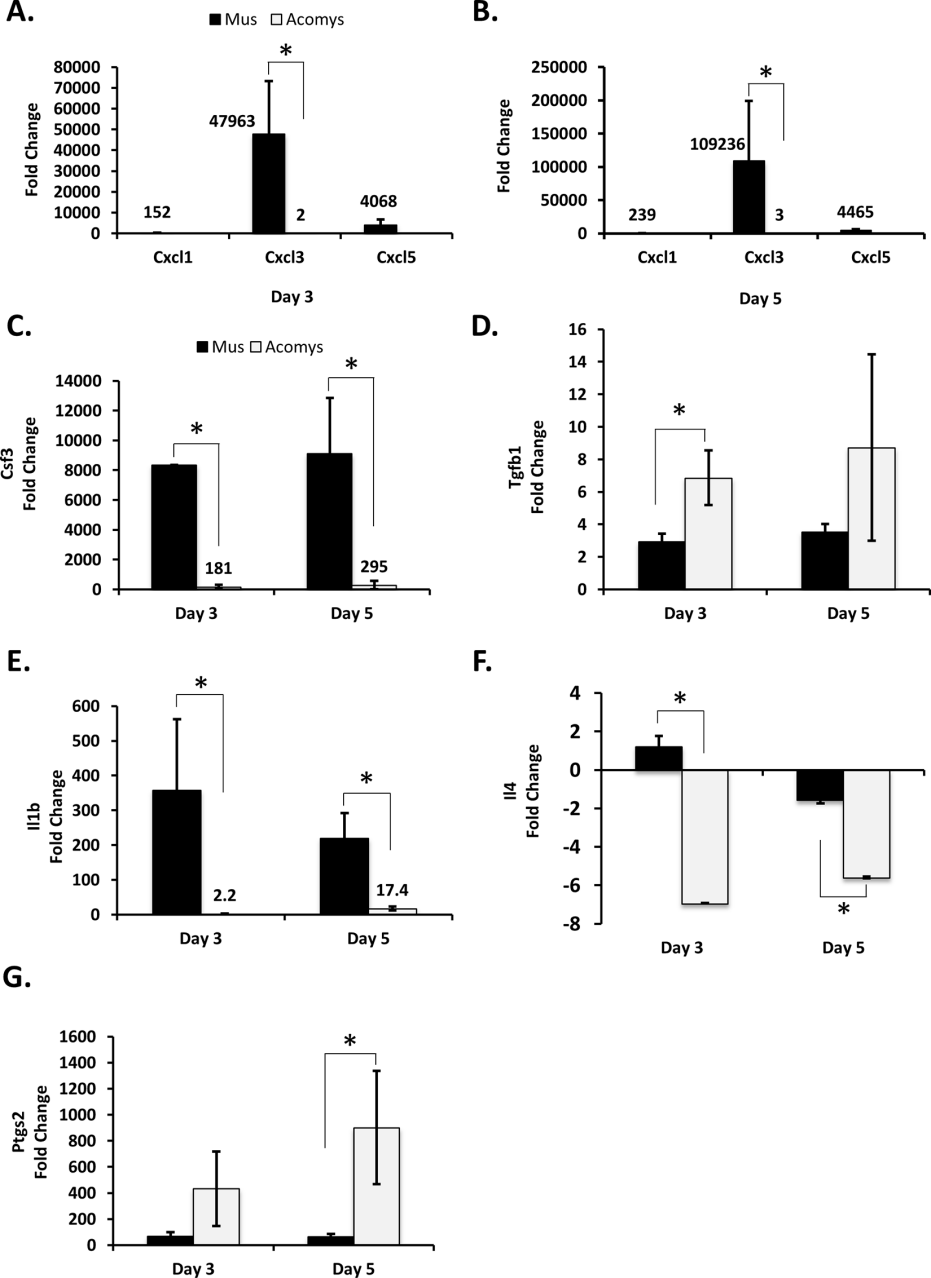
Immune response appears blunted in *Acomys* following wounding. RT-qPCR analysis of day 3 and 5 wounds, compared to normal skin. Data are presented as mean ± SEM. * p ≤ 0.01; n = 3. *Mus* is represented by black bars and *Acomys* is represented by white bars. The Cxcl cytokines, *Cxcl1*, *Cxcl3* and *Cxcl5*, were analyzed for day 3 (A) and day 5 (B) wounds. The remaining genes analyzed were: (C) *Csf3*, (D) *Tgf-β1*(E) *Il1β*, (F) *Il4*, and (G) *Ptgs2*.

The c-x-c motif family of inflammatory chemokines acts by attracting immune cells to the wound site. In *Mus* wounds *Cxcl1*, *Cxcl3* and *Cxcl5* were all significantly upregulated to high degree (from >150 for *Cxcl1* and >4000-fold for *Cxcl3* and *Cxcl5*). In contrast, the increase in *Cxcl3* was relatively modest in *Acomys*, with only a 2 to 3 fold increase in day 3 and 5 wounds compared to normal skin (Fig 1A and 1B). There were no detectable transcripts for *Cxcl1*, and very low levels of transcripts detected for *Cxcl5* in *Acomys* (Fig 1A and 1B).

The glycoprotein granulocyte colony stimulating factor 3 (*Csf 3*) stimulates the production of granulocytes [26], promotes the survival of mature neutrophils [27] and is often used to treat neutropenia [28]. *Csf3* was induced to very high levels of expression in day 3 and 5 wounds in *Mus*. In contrast, the levels of *Csf3* in *Acomys*, while substantially upregulated (~200 to 300 fold) represent approximately 2% of the levels observed in *Mus* (Fig 1C).

*Tgf-β1*(Transforming growth factor beta 1), thought to play a role in nearly all stages of wound healing, was ~3 fold upregulated in *Mus* in day 3 and 5 wounds compared to normal skin. Expression levels of *Tgf-β1* in *Acomys* were approximately twice that observed in *Mus*, *with* a 7-fold increase in expression observed in wounds vs. unwounded skin (Fig 1D).

The interleukin family of secreted cytokines facilitates intercellular communication between immune cells. The pro-inflammatory cytokine *Il1β*, can act pleiotropically to induce a diverse range of effects, such as proliferation and differentiation, lymphocyte activation, angiogenesis and leukocyte attraction [29]. Interleukin 4 (*Il4*) is another pleiotropic cytokine that has been shown to stimulate production of components of the ECM [30–32]. The levels of Interleukins 1β and 4 mRNA were significantly higher in *Mus* than in *Acomys* (Fig 1E and 1F). The expression of *Il1β* was expressed ~350 fold higher and ~220 fold higher in *Mus* day 3 and day 5 wounds relative to unwounded skin, respectively. In *Acomys* the expression of *Il1β* is also elevated in wounded vs. non-wounded skin. However, the magnitude of the elevation in expression of *Il1β* is greatly reduced in *Acomys* relative to *Mus* with *Acomys* day 3 and day 5 wounds exhibiting a 2.2 and 17.4 fold, increase in expression relative to unwounded skin, respectively. (Fig 1E.) There was essentially no change observed in *Il4* expression in *Mus* wounds relative to unwounded skin (<2-fold), while in *Acomys*, *Il4* expression was reduced by 7 and 6.6 fold in day 3 and 5 wounds relative to unwounded skin, respectively (Fig 1F.)

Prostaglandin-endoperoxide synthase 2 (*Ptgs2*, also known as cyclooxygenase-2 (*Cox-2*) is an immediate early response gene that is upregulated immediately after wounding [33], and has been shown to impair wound healing and to promote scar formation [34, 35]. Interestingly, the levels of *Ptgs2* mRNA were significantly higher in *Acomys* than those observed in *Mus* (Fig 1G), with an increase of ~ 70 fold in *Mus* and over 400 fold in *Acomys* observed in both day 3 and 5 wounds.

#### Extracellular matrix

**Fig 2:**
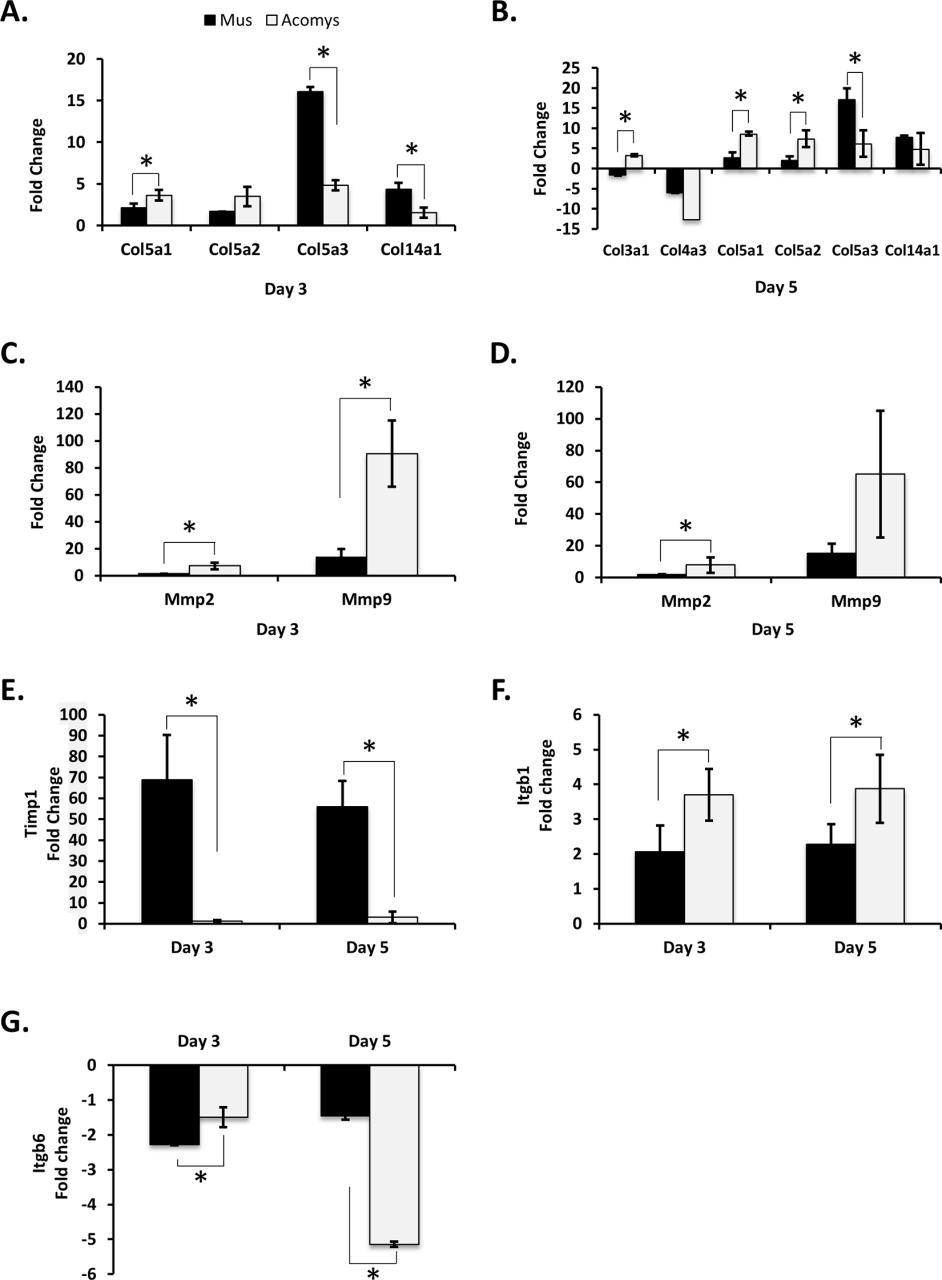
Differences in ECM composition, matrix digestion and cell motility between *Acomys* and *Mus*. RT-qPCR analysis was performed for the following genes: Collagens were analyzed for day 3 (A) and day 5 (B) wounds. *Mmp2* and *9* were analyzed for day 3 (C) and day 5 (D) wounds, *Timp1* (E), *Itgβ1* (F), and *Itgβ6* (G). All labels, symbols and calculations are as those described in Fig 1.

Several genes associated with the organization of the extracellular matrix showed significant differences in mRNA levels between *Mus* and *Acomys* wounds (Fig 2). At days 3 and 5 post wounding, there appears to be a different collagen expression profile between *Mus* and *Acomys* (Fig 2A and 2B). In day 3 wounds *Col5a2* was expressed at significantly higher levels in *Acomys*, compared to unwounded skin, while *Col5a3* and *Col14a1* were significantly higher in *Mus*, compared to unwounded skin (Fig 2A). At 5 days post wounding, both *Col5a1* and *Col5a2* showed significantly higher increases in expression, as compared to unwounded skin, in *Acomys*, while *Col5a3* was higher in *Mus*. *Col4a3* was downregulated in *Mus* and *Acomys* in day 5 wounds, although the difference between the species was not significant (Fig 2B). In addition to components of the ECM, there were also genes involved in ECM degradation which showed differences in their gene expression profiles between *Mus* and *Acomys* (Fig 2C–2E). The matrix metalloproteinases *Mmp2* and *Mmp9* were both significantly upregulated at days 3 and 5 post wounding in the *Acomys* (Fig 2C and 2D). *Mmp2* was upregulated ~ 7.5 fold in both day 3 and 5 wounds in *Acomys*, with no significant changes observed in *Mus*. *Mmp9* was upregulated ~90 fold at day 3, and ~65 fold at day 5 post wounding in *Acomys*. In *Mus*, *Mm9* was upregulated ~13 and 15 fold in day 3 and 5 wounds, respectively (Fig 2C and 2D). Although *Mmp9* was upregulated roughly 15 fold in *Mus* at days 3 and 5, an inhibitor of metalloproteinases, *Timp1*, was upregulated greater than ~50 fold in *Mus*, with no significant increase observed for *Timp1* in *Acomys* (Fig 2E). Taken together, this suggests that ECM degradation is more active in *Acomys* wound healing than in *Mus*.

The Integrin family of proteins plays a key role in cellular binding to the extracellular matrix, as well as relaying information regarding the ECM composition to the cell, and is therefore important in wound healing. For most of the integrins analyzed, there was either no appreciable change in expression in the wounds as compared to normal skin, or there was no detectable level of transcription in at least one time point in the *Acomys* (Table 1). However, integrin β1 (*Itgβ1*) was significantly elevated in day 3 and 5 wounds for both *Mus* and *Acomys*. In *Mus Itgβ1* was upregulated 2.1 fold in day 3 and 2.3 fold in day 5 wounds, while in *Acomys*, *Itgβ1* was upregulated 3.7 fold in day 3 and 3.9 fold in day 5 wounds (Fig 2F). Integrin β6 (*Itgβ6*), which also acts as a potent activator of transforming growth factor (*Tgf*)-beta [36, 37], mRNA levels are lower in both day 3 and 5 wounds for both *Mus* and *Acomys*. In *Mus*, *Itgβ6* is downregulated -2.3 fold in day 3 and -1.5 fold in day 5 wounds. In *Acomys*, *Itgβ6* is downregulated -1.5 fold and -5.1 fold in day 3 and 5 wounds, respectively (Fig 2G). These data suggest that the levels of *Itgβ6* are increasing in *Mus* and decreasing in *Acomys* from days 3 to 5 post wounding, with no change in expression for either species in day 7 and 14 wounds (by microarray analysis).

### Microarray analysis

**Fig 3:**
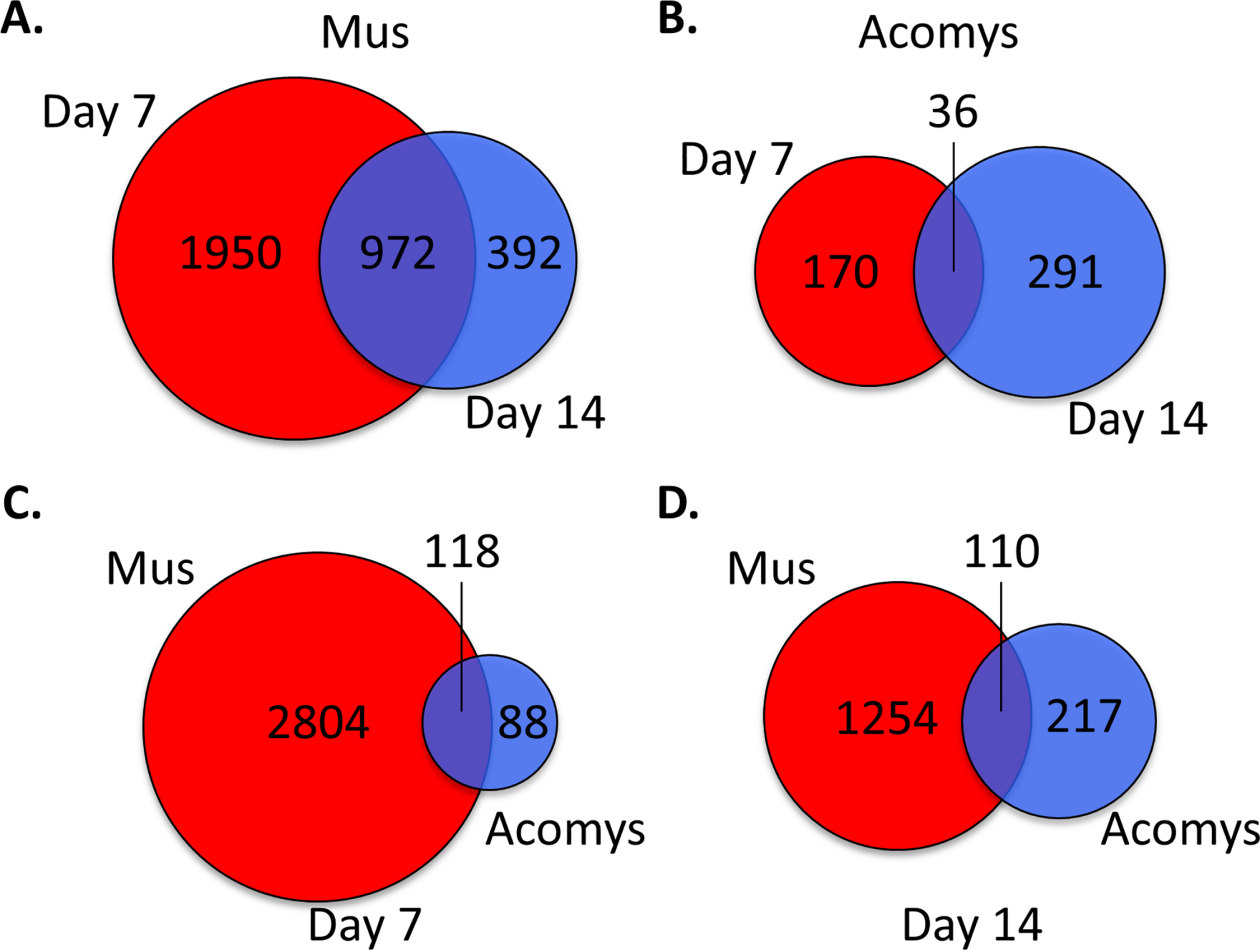
Venn diagram of differentially expressed genes in day 7 and 14 wounds, compared to normal skin. Visual representation of the number of unique differentially genes at days 7 and 14 and those common to both days for (A) *Mus* and (B) *Acomys*. Unique and common differentially expressed genes between *Acomys* and *Mus* for (C) day 7 and (D) day 14. For (A) and (B), day 7 genes are in red, day 14 genes in blue and common genes are the overlap. For (C) and (D) *Mus* genes are in red and *Acomys* genes are in blue and common genes are the overlap.

To examine the mechanisms involved in wound healing, genome wide gene expression analysis of full-depth excisional skin wounds at two additional time points, day 7 and day 14 after wounding, in both *Mus* and *Acomys* were evaluated with Affymetrix GeneChip® microarrays. Changes in gene expression levels were considered significant if the fold change in expression was at least 1.4 fold, in either direction, and with a p-value of significance of ≤ 0.001. At day 7 after wounding there were a total of 2922 genes exhibiting differential expression relative to normal skin in *Mus* (S2 Table). There were 1872 genes that were upregulated and 1050 genes downregulated. At day 14 there were 1364 genes showing statistically significant changes in gene expression compared to normal skin. Of these, 795 were upregulated and 569 were downregulated. Comparing genes with altered expression at both days 7 and 14 post wounding, we find that there were 1950 unique to day 7, 392 unique to day 14, and 972 common to both (Fig 3A).

In *Acomys* there were a total of 206 genes showing differential expression at day 7 after wounding compared to normal skin (S2 Table). Of these, 176 were upregulated and 30 were downregulated. At day 14 there were 327 genes with altered expression compared to normal skin, with 159 genes upregulated and 168 downregulated. Comparing days 7 and 14, there were 170 genes whose change in expression was unique to day 7, 291 unique to day 14, and 30 common to both days 7 and 14 (Fig 3B).

At 7 days post wounding in *Mus*, there was considerable upregulation of genes associated with the inflammatory response, in agreement with our observations by RT-PCR at days 3 and 5. There were a wide array of pro inflammatory genes, including most chemokines and cytokines, whose expression in the wound were increased from ~3 to 90-fold, as well as most interleukins, with interleukin 1 beta (*Il1β*) showing the largest increase at ~145-fold (S2 Table).

While most members of the collagen family were moderately upregulated, as would be expected in a healing wound, collagen 12a1 (*Col12a1*) was increased by nearly 30 fold in *Mus* (this increased expression persisted in the day 14 post wounding samples as well). This was unexpected since there is a lack of evidence in the literature for the role of *Col12a1* in dermal wound healing. We confirmed the high levels of *Col12a1* in *Mus* and lack of *Col12a1* in *Acomys* by immunofluorescence using 2 different antibodies (Brant et al. manuscript under review).

In stark contrast to the high degree of response observed in *Mus*, the overall response to wounding in *Acomys* appears to be reduced, not only in the number of genes affected, but in the magnitude of change as well (from +63 to -2.3 fold). The genes with the largest increase in expression were not those associated with the inflammatory pathways, as observed in *Mus*, but were instead genes associated with the organization of the extracellular-matrix, such as collagens and platelet-derived growth factor binding proteins, as well as peptidases and matrix metalloproteinases (S2 Table).

We recognize that cross-species hybridization could possibly explain the decrease in the number of genes with a change in expression; we therefore performed an analysis of those genes whose change in expression was unique in *Acomys*. That is, these are genes with no detectable level of expression, or no observable change in expression in *Mus* wounds, whose expression is significantly altered in the wound in *Acomys*. Additionally, we also analyzed those genes whose changes in expression were unique to *Mus*. When comparing genes with altered expression at day 7 versus normal skin for both *Mus* and *Acomys*, there are 2804 genes unique to *Mus* and only 88 genes unique to *Acomys*, with 118 genes in common (Fig 3C).

**Fig 4:**
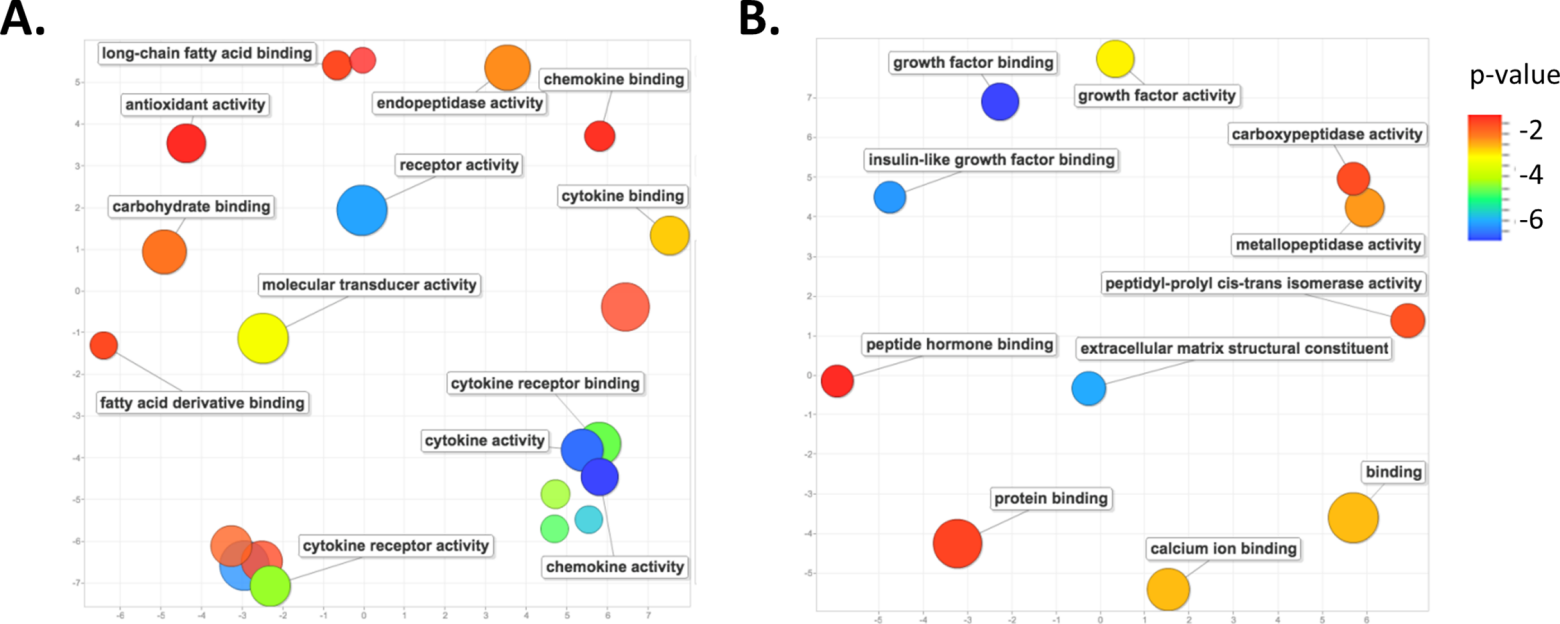
GO term enrichment analysis for differentially expressed genes unique to each species at day 7 post-wounding. Scatter plot representing the summarized GO term analysis of differentially expressed genes at day 7 post wounding in (A) *Mus* and (B) *Acomys*. Semantically similar GO terms are plotted near to each other on the (unit-less) X-Y axes, such that functionally similar terms are located nearby, and more unrelated terms are further apart in space. The size of the individual plots represents the frequency of the GO term in the database so that larger bubbles represent more general terms and smaller bubbles are more specific terms. The color indicates the p-value of enrichment of each GO term.

Those genes whose altered expression is unique to either *Mus* (2804) or *Acomys* (88) could provide insight into the observed difference in wound healing, i.e. scaring in *Mus* versus scar-free wound healing in *Acomys*. We performed an analysis of gene ontology (GO) terms associated with these unique genes. At day 7 post wounding in *Mus* there was a marked enrichment for GO terms associated with the inflammatory pathway, including cytokine receptor activity, chemokine and cytokine activity, cytokine receptor binding, cytokine and chemokine binding, fatty acid and carbohydrate binding, as well as endopeptidase and antioxidant activity (Fig 4A). A pathway enrichment analysis identified the top enriched pathways for genes unique to day 7 *Mus* wounds to be, as expected, those involved in the inflammatory pathways, including cytokine-cytokine receptor interaction, chemokine signaling pathway, Leishmanias, osteoclast differentiation, toll-like receptor signaling pathway and cell adhesion molecules (S3 Table).

In contrast, the top enriched GO terms associated with those genes whose change in expression is unique to day 7 wounds in *Acomys* are those associated with the extracellular matrix reorganization, growth factors and hormone binding, including extracellular matrix structural constituent, metalloproteinase activity, carboxypeptidase activity, growth factor activity and binding, and peptide hormone binding (Fig 4B). The top enriched pathways in *Acomys* were those involved in protein digestion and absorption, extracellular matrix receptor interactions and focal adhesions (S4 Table).

A pathway analysis of the 1364 genes with significantly altered expression in day 14 wounds in *Mus* revealed an enrichment for genes associated with cell adhesion and migration, structural morphogenesis, muscle contraction and contractile fibers and myofibril components, as well as a lingering upregulation of cytokines, chemokines and interleukins (S2 Table). The results of a pathway analysis of the 327 altered genes in *Acomys* were more similar to *Mus* for day 14 than for day 7 wounds. Enriched pathways were observed for contractile fiber components and myofibril genes, as in *Mus*, as well genes associated with tissue development and morphogenesis (S2 Table).

**Fig 5:**
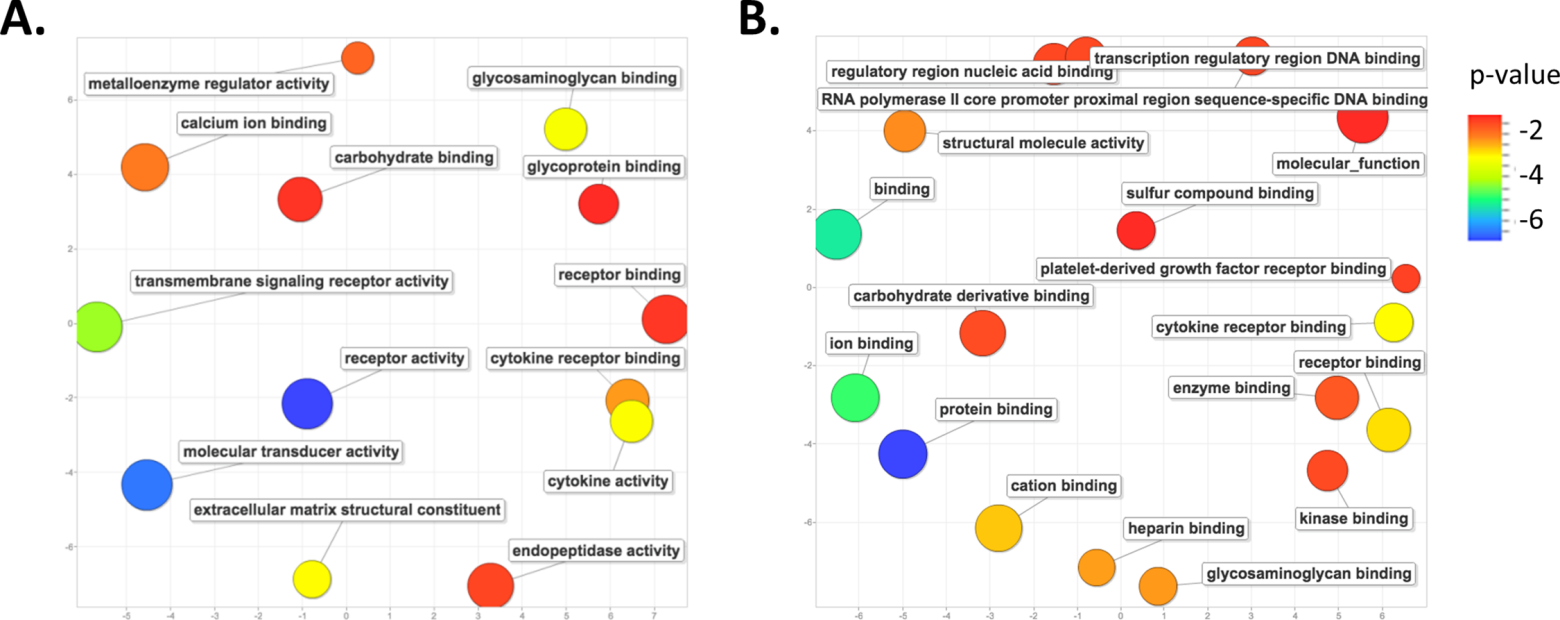
GO term enrichment analysis for differentially expressed genes unique to each species at day 14 post-wounding. Scatter plot representing the summarized GO term analysis of differentially expressed genes at day 14 post wounding in (A) *Mus* and (B) *Acomys*. All labels, symbols and calculations are as those described in Fig 4.

We again performed an analysis of GO terms for those genes whose altered expression is unique to either *Mus* (1254) or *Acomys* (217) at day 14 post-wounding (Fig 3D). The 1254 genes in *Mus* exhibit a persistence of enrichment for genes associated with the inflammation pathway, such as cytokine activity, cytokine receptor binding, and receptor binding and activity. There was also an enrichment for genes involved in the extracellular matrix organization, including metalloenzyme regulator activity and extracellular matrix structural constituent, as well as transmembrane signaling receptor activity and carbohydrate, glycosaminoglycan, glycoprotein and calcium ion binding (Fig 5A). A pathway analysis of these same genes reveals a large number of genes involved in cellular respiration, oxidative phosphorylation, fructose and mannose metabolism, metabolic pathways, as well as the genes involved with the lysosome and focal adhesions (S5 Table).

An enrichment analysis for the GO terms associated with the 217 genes unique to *Acomys* at day 14 wounds reveals genes involved in the regulation of transcription, such as regulatory and transcription regulatory region binding and RNA-polII DNA binding. There was also an enrichment for carbohydrate, protein and ion binding, as well genes involved with cytokine receptor and platelet-derived growth factor receptor binding (Fig 5B). A pathway analysis for these same genes shows enrichment for growth factor pathways, including insulin, VEGF, toll-like receptor, adipocytokine and chemokine signaling pathways (S6 Table).

## Discussion

The recent discovery of the remarkable capacity of *Acomys cahirinus* to regenerate full thickness excisional skin wounds in a scar free manner [21] provides a unique opportunity to compare scar free wound healing with scaring in two adult mammalian tissues. Previous studies were limited to comparing wound healing in adult tissues to fetal tissues, which have a limited ability for scar free healing. Although much has been learned from these studies, there are inherent differences between fetal and adult tissues which imposes limits on these comparative studies and as such, very little has translated into improved clinical outcomes. In this study, we have used RT-PCR and microarray analyses to generate gene expression profiles of full thickness excisional skin wounds in both *Mus* and *Acomys* to compare scar free wound healing to scaring in adult tissue and to determine if there are any similarities to scar free healing observed in fetal tissues. We have identified changes in gene expression patterns for several aspects of wound healing between *Mus* and *Acomys*, particularly for those genes involved in the inflammatory pathway and the deposition and digestion of the extracellular matrix, and have identified similarities to fetal wounds.

There are several notable differences between fetal and adult wounds, which are predominately in the inflammatory pathway and the collagen profile of the ECM and in expression levels of various growth factors. Previous studies have shown that the inflammatory response is quite blunted in fetal wounds [4, 5]. Additionally, it has been shown that ectopically induced inflammation in fetal wounds increases fibrosis and results in scar formation [38, 39]. Our data indicates that wounding initiates a strong inflammatory response in *Mus*, typical of the adult mammalian response, while the inflammatory response in *Acomys* appears to be modest, similar to fetal wounds.

Immediately after wounding platelets enter the wound bed and release *Pdgfa* and *Tgf-β*1/2 [40–42]. Studies have shown that *Pdgfa* and *Tgf-β*1/2 levels are lower in fetal compared to adult wounds [43]. In contrast to fetal wounds, our data indicate no difference in *Pdgfa* expression in day 3 and 5 wounds, and an increase in *Tgf-β*1 expression in *Acomys*.

Shortly after platelets enter the wound, neutrophils are recruited by neutrophil attracting chemokines and activated by neutrophil activating cytokines (reviewed extensively in [44–46]). Studies have shown that neutrophil levels are higher in adult wounds compared to fetal wounds [9]. Counts of circulating leukocytes, from non-wounded animals, revealed that neutrophils are present in *Acomys* at only 20% the levels observed in *Mus* (Brant et al. manuscript under review). The impact of reduced circulating neutrophil levels is unknown at this time but certainly warrants further study. By day 2 after wounding, macrophages constitute the predominant blood-derived cell type in the wound bed and initiate release of pro inflammatory cytokines and growth factors [47]. Our data show a striking difference in the expression levels of several genes thought to play important roles in inflammation. The expression of the chemokine *Cxcl3* is only slighter higher in *Acomys* wounds, compared to normal skin, and *Cxcl1* and *Cxcl5* are barely detectable by RT-PCR, in contrast to the high levels of induction observed in *Mus*. As the c-x-c like family of chemokines is expressed by macrophages and neutrophils, this observed difference in expression could in part be explained by the observed reduction in circulating neutrophils in *Acomys*. Additionally, the induction of granulocyte stimulating factor *Csf3* mRNA is much lower in *Acomys* as well, suggesting an overall diminished role of neutrophils in *Acomys*. We have additional data that shows that while macrophages are present in *Acomys* in very early wounds, they are confined to the underlying fascial connective tissue and are not present in the wound bed at later time points (Brant et al. manuscript in review). The increased upregulation of *Tgf-β*1 is intriguing in light of the seemingly blunted immune response observed in *Acomys*, given its role in promoting inflammation, among others, during wound healing. It is also interesting to note that while differences have been observed for various growth factors between fetal and adult wounds [34, 48–50], our data indicate that there were no statistically significant changes observed in expression levels of the growth factors *Csf2*, *Ctgf*, *Egf*, *Fgf2*, *Fgf10* and *Vegfa* between *Mus* and *Acomys* wounds (S1 Table).

Interleukin 4 expression is significantly downregulated in *Acomys*, while there is no significant change in expression for *Mus*. As *Il4* is thought to promote alternative activation of macrophages [51–53], the downregulation of *Il4* seems to run counter to the observed blunted inflammatory response. *Il4* has also been shown to be involved in the stimulation of production of components of the ECM [30–32]. Perhaps lower levels of *Il4* prevent an overproduction of collagen formation and may help to prevent scar formation. Interleukin 10 (*Il10*) is known to be a potent anti-inflammatory pleiotropic cytokine produced by a number of cell types, including T cells, B cells, monocytes and alternatively activated macrophages (reviewed extensively in [54] and [55]). While *Il10* is upregulated approximately 6 fold in *Mus* day 3 and 5 wounds, there is no detectable expression of *Il10* in *Acomys*. This could be due to an absence of those cells that produce *Il10* in the wound, as we have shown with F4/80 and cd206 +ve macrophages (Brant et al. manuscript in review). Alternatively, it may be that the *Mus Il10* PCR primers do not efficiently anneal to *Acomys Il10* sequence. Given the importance of *Il10* function in dampening the inflammatory response, this will need to be studied further going forward. *Acomys* also exhibits much lower levels of *Il1β* expression in wounds than in *Mus*. *Il1β* is a potent proinflammatory cytokine produced by monocytes and activated macrophages [56, 57]. *Il1β* is also known to induce *Ptgs2* expression [58], although the expression of *Ptgs2* is much higher in *Acomys* wounds compared to normal skin than in *Mus* wounds. The high levels of *Ptgs2* in *Acomys* wounds is interesting, in that it also seems contrary to data suggesting *Ptgs2* levels are correlated with scar formation in skin wounds. Wilgus et al. showed that the *Ptgs2* inhibitor Celecoxib® decreased inflammation in incisional wounds and reduced scar formation [35] and have also shown that high levels of *Ptgs2* promote scarring and delay wound closure in fetal wounds [34].

The collagen composition of fetal skin has been shown to differ from adult skin [12–14], with fetal skin having higher levels of collagen type III and V than adult skin, among other differences. The expression of collagens in *Acomys* wounds suggests that they have a profile similar to fetal skin, with higher levels of expression for collagens 3a1, 5a1, 5a2, and lower levels of expression of collagens 5a3 and 14a1. In addition to differences in the composition of the ECM, there are notable differences in the expression of matrix metalloproteinases involved in the degradation of the ECM. Fetal skin wounds have been shown to have a higher matrix metalloproteinase to tissue inhibitor of metalloproteinase ratio [16, 17]. Similar to fetal wounds, *Acomys* wounds have significantly higher levels of expression of *Mmp2* and *Mmp9*, and lower levels of expression of *Timp1* in day 3 and 5 wounds compared to *Mus*. This suggests that there is a higher turnover of ECM components and an increase in cell migration through the wound bed in *Acomys*. Fetal wounds have also been shown to have essentially no myofibroblasts, which are thought to interact with the ECM and aid in wound closure through contraction, while both scaring fetal and adult wounds have high levels of contractile myofibroblasts appearing 7 days post wounding [59]. Although *Acomys* wounds heal in a scar-free manner, similar to fetal wounds, our data indicate there is no difference in the expression of smooth muscle actin, a marker of myofibroblasts, between *Mus* and *Acomys* wounds from days 3, 5 (S1 Table), 7 and 14 (S2 Table).

We considered the possibility that some of the observed differences in expression could be the result of inefficient cross species hybridization of oligos used in the RT-PCR arrays. Although the primers used in the arrays were designed for *Mus*, there was detectable amplification for 54/84 genes in *Acomys*, compared to 79/84 for *Mus*. This could be due to the fact that not all genes from the array are expressed in skin or wounds in both species. Alternatively, some genes may have sufficiently diverged from the mouse, such that *Mus* specific primers do not anneal. In either case, the majority of genes have detectable levels of amplification, suggesting a fairly high degree of conservation, at least for coding regions. In order to confirm that we are in fact amplifying the correct mRNA, we have cloned and sequenced the *Tgf-β*1, *Timp1* and *Mmp9* RT-PCR amplicons from *Acomys*. A BLAST using the resulting sequences against the *Mus* transcript database reveals high identity matches for all three sequences (S1A–S1C Fig). The percent identity for *Tgf-β*1 and *Timp1* was 98% and 100%, respectively. Although the percent identity for *Mmp9* was 74% for *Mus*, there were higher identity matches in other rodent species, e.g. *Peromyscus maniculatus* (Deer mouse, 91%).

Microarray analysis was performed for both *Acomys* and *Mus* wounds at day 7 and 14 post wounding. The expression data for day 7 wounds are similar to the day 3 and 5 wound data, confirming the observation that the inflammatory response in *Acomys* is quite blunted and further validates our RT-PCR data. We appreciate the fact that the reduced number of differentially expressed genes observed in *Acomys* wounds, as compared to *Mus*, could be an issue of cross-species hybridization, as we used microarrays designed for use with *Mus* RNA. Therefore, part of our analysis was focused on those genes whose changes in expression are unique to *Acomys*. We felt that these would be of the highest confidence, giving the limits of the technology, and could provide the most significant biological insights into scar free healing. Pathway analysis and GO term enrichment analysis of these unique genes gave the same results as analyses using the whole dataset, i.e. using both unique and common sets of differentially expressed genes, suggesting that the observed enriched pathways are largely driven by those genes which are uniquely differentially expressed in each species and not necessarily by those whose change is common to both species.

In summary, the data presented here provide insights into scar free healing of full-thickness excisional wounds of *Acomys*, and provides a starting point of potential gene candidates that may be further studied, in hopes of devising potential strategies for improved clinical outcomes in preventing scarring in humans.

## Supporting Information

S1 Fig BLAST search results of *Acomys* RT-PCR amplicons against *Mus* transcript database. BLAST results of cloned and sequenced *Acomys* RT-PCR amplicons against the *Mus* transcript database for (A) *Tgf-β1*, (B) *Timp1*, and (C) *Mmp9*.
(PDF)

S1 Table Genes analyzed by RT-PCR in day 3 and 5 wounds. List of genes analyzed by Wound-Healing RT^2^ Profiler Array in day 3 and 5 wounds, compared to normal skin, within each species. Bold entries are those with a p-value ≤0.01.
(DOCX)

S2 Table Results of microarray analysis. Tables of p-values and fold-change in expression with each analysis in its own worksheet.
(XLSX)

S3 Table Pathway analysis of *Mus* day 7 wounds. Pathway analysis of differentially expressed genes between day 7 wounds and normal skin in *Mus*.
(DOCX)

S4 Table Pathway analysis of *Acomys* day 7 wounds. Pathway analysis of differentially expressed genes between day 7 wounds and normal skin in *Acomys*.
(DOCX)

S5 Table Pathway analysis of *Mus* day 14 wounds. Pathway analysis of differentially expressed genes between day 14 wounds and normal skin in *Mus*.
(DOCX)

S6 Table Pathway analysis of *Acomys* day 14 wounds. Pathway analysis of differentially expressed genes between day 14 wounds and normal skin in *Acomys*.
(DOCX)
