# Supplementary material for: A Comparative Analysis of Gene Expression Profiles during Skin Regeneration in Mus and Acomys
Source: PLoS One. 2015 Nov 25;10(11):e0142931. doi: 10.1371/journal.pone.0142931 (PMC4659537; doi:10.1371/journal.pone.0142931)
Supplement: S1 Fig — BLAST results of cloned and sequenced Acomys RT-PCR amplicons against the Mus transcript database for (A) Tgf-β1, (B) Timp1, and (C) Mmp9. (PDF) [file pone.0142931.s001.pdf]

A

Mus musculus transforming growth factor, beta 1 (Tgfb1), mRNA

Sequence ID: ref|NM_011577.1| **Length:** 2094 **Number of Matches:** 1

Range 1: 1506 to 1568 GenBank Graphics ▼ Next Match ▲ Previous Match

Score	Expect	Identities	Gaps	Strand
111 bits(60)	4e-23	62/63(98%)	0/63(0%)	Plus/Plus

```
Query  1     AATACAGGGCTTTCGATTCAGTGCTCACTGCTCTTGTGACAGCAAAGATAACAAACTCCA  60
             ||||||||||||||||||||| ||||||||||||||||||||||||||||||||||||||
Sbjct  1506  AATACAGGGCTTTCGATTCAGCGCTCACTGCTCTTGTGACAGCAAAGATAACAAACTCCA  1565

Query  61    CGT  63
             |||
Sbjct  1566  CGT  1568
```

B

Mus musculus tissue inhibitor of metalloproteinase 1 (Timp1), transcript variant 3, mRNA

Sequence ID: ref|NM_001294280.2| **Length:** 825 **Number of Matches:** 1

Range 1: 613 to 695 GenBank Graphics ▼ Next Match ▲ Previous Match

Score	Expect	Identities	Gaps	Strand
154 bits(83)	1e-35	83/83(100%)	0/83(0%)	Plus/Plus

```
Query  1    CTCGTGGGCTCTGAGGACTACCAGAGCCGTCACTTTGCTTGCCTGCCACGGAATCCAGGC  60
            ||||||||||||||||||||||||||||||||||||||||||||||||||||||||||||
Sbjct  613  CTCGTGGGCTCTGAGGACTACCAGAGCCGTCACTTTGCTTGCCTGCCACGGAATCCAGGC  672

Query  61   TTGTGCACCTGGAGATCCCTTGG  83
            |||||||||||||||||||||||
Sbjct  673  TTGTGCACCTGGAGATCCCTTGG  695
```

C

PREDICTED: Mus musculus matrix metallopeptidase 9 (Mmp9), transcript variant X1, mRNA

Sequence ID: ref|XM_006498861.2| **Length:** 3220 **Number of Matches:** 1

Range 1: 2163 to 2266 GenBank Graphics ▼ Next Match ▲ Previous Match

Score	Expect	Identities	Gaps	Strand
77.0 bits(84)	3e-11	77/104(74%)	21/104(20%)	Plus/Plus

```
Query  1     GGCAAATTCTTCTGGCGTGTGAATTTCCA-----AGG----------------GGAGGTG  39
             |||||||||||||||||||||| ||||||     |||                 ||||||
Sbjct  2163  GGCAAATTCTTCTGGCGTGTGAGTTTCCAAAATGAGGTGAACAAGGTGGACCATGAGGTG  2222

Query  40    AACCAGGTGGACCACGTCGGGTATGTGACCTACGACCTCCTGCA  83
             |||||||||||| |||| || || ||||||||||||||||||||
Sbjct  2223  AACCAGGTGGACGACGTGGGCTACGTGACCTACGACCTCCTGCA  2266
```
