# Supplementary material for: A Comparative Analysis of Gene Expression Profiles during Skin Regeneration in Mus and Acomys
Source: PLoS One. 2015 Nov 25;10(11):e0142931. doi: 10.1371/journal.pone.0142931 (PMC4659537; doi:10.1371/journal.pone.0142931)
Supplement: S1 Table — List of genes analyzed by Wound-Healing RT2 Profiler Array in day 3 and 5 wounds, compared to normal skin, within each species. Bold entries are those with a p-value ≤0.01. (DOCX) [file pone.0142931.s002.docx]

**Supplemental Table 1. Genes Analyzed by RT-PCR in day 3 and 5 wounds.**

|  | ***Mus musculus*** | | ***Acomys cahirinus*** | |
| --- | --- | --- | --- | --- |
| **Gene** | **Day 3 vs 0** | **Day 5 vs 0** | **Day 3 vs 0** | **Day 5 vs 0** |
| **Acta2** | -1.146 | 1.513 | 1.146 | -1.609 |
| **Actc1** | 2.408 | **12.429** | **26.960** | 5.313 |
| **Angpt1** | 1.940 | 3.263 | -1.130 | 1.184 |
| **Ccl12** | 2.254 | 2.005 | N/A | N/A |
| **Ccl7** | **7.142** | **11.577** | N/A | N/A |
| **Cd40lg** | 1.246 | -5.270 | N/A | N/A |
| **Cdh1** | -2.176 | -2.021 | -1.071 | -1.191 |
| **Col14a1** | **4.364** | **7.799** | 1.552 | 4.846 |
| **Col1a1** | 1.795 | 2.928 | 1.334 | 5.191 |
| **Col1a2** | -2.054 | -1.512 | 1.511 | 2.453 |
| **Col3a1** | -2.144 | -1.551 | 1.528 | **3.313** |
| **Col4a1** | 1.569 | 2.202 | 1.668 | 2.116 |
| **Col4a3** | -10.323 | **-5.921** | 1.561 | -12.627 |
| **Col5a1** | 2.088 | 2.805 | **3.623** | **8.626** |
| **Col5a2** | 1.671 | 2.118 | **3.489** | **7.405** |
| **Col5a3** | **16.050** | **17.202** | **4.811** | 6.196 |
| **Csf2** | 13.388 | 34.471 | -2.768 | -1.273 |
| **Csf3** | **8369.125** | **9126.401** | **181.376** | **294.590** |
| **Ctgf** | -2.026 | -2.358 | -1.262 | 18.473 |
| **Ctnnb1** | 1.196 | 1.732 | 3.667 | 6.248 |
| **Ctsg** | **-8.594** | **-2.514** | 2.321 | N/A |
| **Ctsk** | -1.660 | 1.195 | -23.454 | N/A |
| **Ctsl** | 1.976 | 1.892 | 2.160 | 2.579 |
| **Cxcl1** | **151.834** | **239.431** | N/A | N/A |
| **Cxcl11** | -1.685 | -1.294 | 1.462 | 1.843 |
| **Cxcl3** | **47963.253** | **109235.609** | 1.775 | 3.308 |
| **Cxcl5** | **4067.714** | **4464.773** | N/A | N/A |
| **Egf** | -2.099 | -1.828 | -1.145 | N/A |
| **Egfr** | -1.560 | -1.525 | -2.500 | -1.641 |
| **F13a1** | 2.133 | 1.994 | 2.960 | 1.045 |
| **F3** | 1.314 | 1.538 | N/A | N/A |
| **Fga** | N/A | N/A | N/A | N/A |
| **Fgf10** | -2.116 | -6.446 | 3.017 | -2.676 |
| **Fgf2** | -2.445 | -2.325 | 2.126 | **4.205** |
| **Fgf7** | 1.901 | **2.918** | N/A | N/A |
| **Hbegf** | 2.805 | 3.433 | N/A | N/A |
| **Hgf** | 3.851 | **5.408** | N/A | N/A |
| **Ifng** | -1.180 | **-2.665** | N/A | N/A |
| **Igf1** | -1.152 | 1.434 | -1.352 | 1.024 |
| **Il10** | **6.664** | **6.198** | N/A | N/A |
| **Il1b** | **358.635** | **220.279** | 2.231 | 17.350 |
| **Il2** | -5.425 | 2.130 | 18.924 | 8.591 |
| **Il4** | 1.208 | -1.557 | **-6.935** | **-5.590** |
| **Il6** | 14.650 | 8.339 | N/A | N/A |
| **Il6st** | 1.032 | 1.335 | N/A | N/A |
| **Itga1** | -1.051 | 1.497 | 4.846 | 1.960 |
| **Itga2** | 1.205 | 1.134 | -8.510 | N/A |
| **Itga3** | 1.119 | -1.104 | 1.123 | N/A |
| **Itga4** | 1.750 | 2.060 | N/A | N/A |
| **Itga5** | 6.290 | 4.885 | N/A | 6.925 |
| **Itga6** | -1.123 | -1.820 | 2.236 | 1.677 |
| **Itgav** | 1.065 | 1.344 | 2.397 | 1.538 |
| **Itgb1** | 2.062 | 2.273 | **3.700** | **3.875** |
| **Itgb3** | **3.250** | **4.290** | **4.363** | **9.454** |
| **Itgb5** | 1.373 | 2.179 | 1.231 | -1.097 |
| **Itgb6** | **-2.272** | -1.449 | -1.494 | **-5.143** |
| **Mapk1** | -1.294 | -1.098 | -1.298 | -1.093 |
| **Mapk3** | -1.182 | 1.228 | N/A | N/A |
| **Mif** | **3.030** | **3.755** | **1.566** | 1.807 |
| **Mmp1a** | -1.763 | -2.066 | 1.511 | N/A |
| **Mmp2** | 1.158 | 1.743 | **7.223** | **7.881** |
| **Mmp7** | N/A | N/A | 2.349 | -10.371 |
| **Mmp9** | **13.534** | **14.893** | **90.509** | **65.085** |
| **Pdgfa** | -1.412 | -1.344 | -2.875 | N/A |
| **Plat** | **3.648** | 4.283 | 1.927 | **3.433** |
| **Plau** | 3.740 | **3.786** | N/A | N/A |
| **Plaur** | **48.848** | **62.479** | -1.337 | N/A |
| **Plg** | N/A | N/A | N/A | N/A |
| **Pten** | -1.809 | -1.734 | 1.876 | 1.848 |
| **Ptgs2** | **70.466** | **67.940** | **433.720** | **902.276** |
| **Rac1** | -1.638 | -1.663 | 1.564 | 1.952 |
| **Rhoa** | 1.176 | 1.400 | 2.808 | 3.627 |
| **Serpine1** | **233.382** | **275.948** | N/A | N/A |
| **Stat3** | 1.396 | 1.562 | 2.434 | 1.711 |
| **Tagln** | -1.890 | -1.565 | -2.328 | 1.620 |
| **Tgfa** | -1.132 | -1.431 | N/A | N/A |
| **Tgfb1** | **2.937** | **3.545** | **6.862** | **8.716** |
| **Tgfbr3** | 2.113 | 2.755 | 2.685 | -1.489 |
| **Timp1** | **68.761** | **56.027** | 1.294 | 3.095 |
| **Tnf** | **27.552** | **29.862** | **54.254** | **31.358** |
| **Vegfa** | **4.285** | **6.506** | **5.741** | **7.545** |
| **Vtn** | 1.369 | 1.839 | N/A | N/A |
| **Wisp1** | **10.296** | **15.814** | 6.127 | 9.138 |
| **Wnt5a** | 1.285 | 1.446 | 1.357 | 1.164 |

List of genes analyzed by Wound-Healing RT^2^ Profiler Array in day 3 and 5 wounds, compared to normal skin, within each species. Bold entries are those with a p-value ≤0.01.
