# Supplementary material for: A Comparative Analysis of Gene Expression Profiles during Skin Regeneration in Mus and Acomys
Source: PLoS One. 2015 Nov 25;10(11):e0142931. doi: 10.1371/journal.pone.0142931 (PMC4659537; doi:10.1371/journal.pone.0142931)
Supplement: S3 Table — Pathway analysis of differentially expressed genes between day 7 wounds and normal skin in Mus. (DOCX) [file pone.0142931.s004.docx]

**Supplemental Table 3. Pathway Analysis of *Mus* day 7 wounds**

| **Pathway Name** | **# of Genes** | **p-value** | **Gene Symbol** |
| --- | --- | --- | --- |
| **Cytokine-Cytokine Receptor Interaction** | 89 | 5.46e-26 | Acvr2b; Bmp2; Ccl3; Ccl4; Ccl5; Ccl6; Ccl7; Ccl8; Ccl9; Ccr1; Ccr2; Ccr3; Ccr5; Ccr7; Cd27; Cd40; Csf1; Csf1r; Csf2ra; Csf2rb2; Csf3r; Cxcl1; Cxcl10; Cxcl11; Cxcl13; Cxcl14; Cxcl16; Cxcl2; Cxcl5; Cxcl9; Cxcr2; Cxcr4; Cxcr6; Egf; Fas; Fasl; Figf; Flt3; Hgf; Ifnar1; Ifnar2; Il10ra; Il10rb; Il12a; Il15; Il17ra; Il18rap; Il1a; Il1b; Il1r1; Il1r2; Il1rap; Il20ra; Il20rb; Il21r; Il22ra2; Il23r; Il2ra; Il2rb; Il2rg; Il4ra; Il6; Il7r; Inhba; Kdr; Lepr; Ltb; Osm; Pdgfd; Pdgfrb; Pf4; Ppbp; Prlr; Tgfb1; Tgfbr1; Tnf; Tnfrsf10b; Tnfrsf14; Tnfrsf19; Tnfrsf1b; Tnfrsf8; Tnfrsf9; Tnfsf10; Tnfsf13b; Tnfsf14; Tnfsf9; Vegfa; Vegfb; Vegfc |
| **Chemokine Signaling Pathway** | 68 | 3.33e-21 | Adcy1; Adcy4; Adcy7; Akt3; Arrb2; Ccl2; Ccl3; Ccl4; Ccl5; Ccl6; Ccl7; Ccl8; Ccl9; Ccr1; Ccr2; Ccr3; Ccr5; Ccr7; Cdc42; Cxcl1; Cxcl10; Cxcl11; Cxcl13; Cxcl14; Cxcl16; Cxcl2; Cxcl5; Cxcl9; Cxcr2; Cxcr4; Cxcr6; Dock2; Elmo1; Fgr; Gnai1; Gnai2; Gnb4; Gng11; Gngt2; Grk5; Hck; Hras1; Jak2; Jak3; Lyn; Ncf1; Nfkbia; Pard3; Pf4; Pik3cd; Pik3cg; Pik3r5; Plcb1; Plcb2; Ppbp; Prex1; Prkcd; Prkcz; Pxn; Rac2; Rap1a; Rap1b; Stat1; Stat2; Stat3; Tiam1; Vav1; Was |
| **Leishmaniasis** | 33 | 1.14e15 | C3; Cyba; Fcgr1; Fcgr3; Fcgr4; Fos; H2-Aa; H2-Ab1; Il12a; Il1a; Il1b; Irak4; Itga4; Itgam; Itgb1; Itgb2; Jak1; Jak2; Jun; Marcksl1; Myd88; Ncf1; Ncf2; Ncf4; Nfkbia; Nos2; Ptgs2; Ptpn6; Stat1; Tgfb1; Tlr2; Tlr4; Tnf |
| **Osteoclast Differentiation** | 46 | 1.22e-15 | Akt3; Btk; Csf1; Csf1r; Cyba; Cybb; Fcgr1; Fcgr2b; Fcgr3; Fcgr4; Fhl2; Fos; Fosl2; Fyn; Ifnar1; Ifnar2; Il1a; Il1b; Il1r1; Irf9; Itgb3; Jak1; Jun; Junb; Lcp2; Lilra6; Lilrb3; Ncf1; Ncf2; Ncf4; Nfkb2; Nfkbia; Pik3cd; Pik3cg; Pik3r5; Sfpi1; Sirpa; Socs3; Stat1; Stat2; Syk; Tgfb1; Tgfbr1; Tnf; Trem2; Tyrobp |
| **Toll-Like Receptor Signaling Pathway** | 40 | 9.11e-14 | Akt3; Casp8; Ccl3; Ccl4; Ccl5; Cd14; Cd40; Cd80; Cd86; Cxcl10; Cxcl11; Cxcl9; Fos; Ifnar1; Ifnar2; Il12a; Il1b; Il6; Irak4; Irf5; Irf7; Jun; Myd88; Nfkbia; Pik3cd; Pik3cg; Pik3r5; Ripk1; Spp1; Stat1; Tbk1; Ticam2; Tlr1; Tlr2; Tlr4; Tlr6; Tlr7; Tlr8; Tlr9; Tnf |
| **Cell Adhesion Molecules (CAMs)** | 48 | 9.07e-13 | Cadm3; Cd22; Cd226; Cd274; Cd4; Cd40; Cd80; Cd86; Cdh2; Cdh4; Cdh5; Cldn23; Cldn3; Cldn8; Esam; H2-Aa; H2-Ab1; H2-M3; H2-M9; H2-Q10; Icam1; Icam2; Icos; Itga4; Itga9; Itgal; Itgam; Itgb1; Itgb2; Mpz; Ncam2; Nlgn2; Ocln; Pdcd1lg2; Pecam1; Ptprc; Ptprf; Ptprm; Pvrl3; Sdc2; Sdc3; Sdc4; Sell; Selp; Selplg; Siglec1; Vcam1; Vcan |

Pathway analysis of differentially expressed genes between day 7 wounds and normal skin in *Mus*.
