# Supplementary material for: A Comparative Analysis of Gene Expression Profiles during Skin Regeneration in Mus and Acomys
Source: PLoS One. 2015 Nov 25;10(11):e0142931. doi: 10.1371/journal.pone.0142931 (PMC4659537; doi:10.1371/journal.pone.0142931)
Supplement: S4 Table — Pathway analysis of differentially expressed genes between day 7 wounds and normal skin in Acomys. (DOCX) [file pone.0142931.s005.docx]

**Supplemental Table 4. Pathway Analysis of *Acomys* day 7 wounds**

| **Pathway Name** | **# of Genes** | **p-value** | **Entrez Gene ID** |
| --- | --- | --- | --- |
| **Protein Digestion and Absorbtion** | 4 | 0.0002 | Col1a1; Col1a2; Ace2; Eln |
| **ECM-Receptor Interactions** | 3 | 0.0035 | Col1a1; Col1a2; Sv2b |
| **Focal Adhesion** | 4 | 0.0054 | Col1a1; Col1a2; Pdgfc; Myl7 |

Pathway analysis of differentially expressed genes between day 7 wounds and normal skin in *Acomys*.
