# Supplementary material for: A Comparative Analysis of Gene Expression Profiles during Skin Regeneration in Mus and Acomys
Source: PLoS One. 2015 Nov 25;10(11):e0142931. doi: 10.1371/journal.pone.0142931 (PMC4659537; doi:10.1371/journal.pone.0142931)
Supplement: S5 Table — Pathway analysis of differentially expressed genes between day 14 wounds and normal skin in Mus. (DOCX) [file pone.0142931.s006.docx]

**Supplemental Table 5. Pathway Analysis of *Mus* day 14 wounds**

| **Pathway Name** | **# of Genes** | **p-value** | **Entrez Gene ID** |
| --- | --- | --- | --- |
| **Parkinson’s Disease** | 34 | 2.27e-15 | Apaf1; Atp5a1; Atp5j; Casp3; Cox4i1; Cox5a; Cox6a2; Cox7a1; Cox7a2; Cox8b; Cyc1; Cycs; Htra2; LOC100046079; Lrrk2; Ndufa2; Ndufa6; Ndufa9; Ndufb4; Ndufb8; Ndufb9; Ndufs2; Ndufs6; Ndufs7; Ndufv1; Park7; Pink1; Sdhb; Sdhc; Slc25a4; Uba7; Uqcrc1; Uqcrc2; Vdac1 |
| **Alzheimer’s Disease** | 37 | 5.62e-14 | Apaf1; Atp5a1; Atp5j; Cacna1c; Cacna1d; Capn2; Casp12; Casp3; Cox4i1; Cox5a; Cox6a2; Cox7a1; Cox7a2; Cox8b; Cyc1; Cycs; Gapdh; Itpr1; Itpr3; LOC100046079; Mapt; Ndufa2; Ndufa6; Ndufa9; Ndufb4; Ndufb8; Ndufb9; Ndufs2; Ndufs6; Ndufs7; Ndufv1; Nos1; Plcb2; Sdhb; Sdhc; Uqcrc1; Uqcrc2 |
| **Huntington’s Disease** | 34 | 2.69e-11 | Apaf1; Atp5a1; Atp5j; Casp3; Cox4i1; Cox5a; Cox6a2; Cox7a1; Cox7a2; Cox8b; Cyc1; Cycs; Gpx1; Itpr1; LOC100046079; Ndufa2; Ndufa6; Ndufa9; Ndufb4; Ndufb8; Ndufb9; Ndufs2; Ndufs6; Ndufs7; Ndufv1; Plcb2; Ppargc1a; Sdhb; Sdhc; Slc25a4; Tgm2; Uqcrc1; Uqcrc2; Vdac1 |
| **Oxidative Phosphorylation** | 27 | 7.16e-10 | Atp5a1; Atp5j; Atp6ap1; Atp6v0d2; Cox4i1; Cox5a; Cox6a2; Cox7a1; Cox7a2; Cox8b; Cyc1; LOC100046079; Ndufa2; Ndufa6; Ndufa9; Ndufb4; Ndufb8; Ndufb9; Ndufs2; Ndufs6; Ndufs7; Ndufv1; Sdhb; Sdhc; Tcirg1; Uqcrc1; Uqcrc2 |
| **Lysosome** | 24 | 8.95e-09 | Ap1m1; Atp6ap1; Atp6v0d2; Cd63; Cd68; Ctsb; Ctsc; Ctss; Ctsz; Galns; Gba; Gns; Gusb; Igf2r; Lgmn; Lipa; M6pr; Man2b1; Manba; Naglu; Pla2g15; Psap; Slc11a1; Tcirg1 |
| **Fructose and Mannose Metabolism** | 12 | 1.37e-07 | Akr1b8; Aldoa; Fbp2; Fuk; Gmppa; Gmppb; Hk3; Pfkfb1; Pfkfb4; Pfkl; Pfkm; Tpi1 |
| **Cardiac Muscle Contraction** | 17 | 3.59e-07 | Atp1a2; Atp1b1; Cacna1c; Cacna1d; Cacnb1; Cacng1; Cox4i1; Cox5a; Cox6a2; Cox7a1; Cox7a2; Cox8b; Cyc1; LOC100046079; Tpm4; Uqcrc1; Uqcrc2 |
| **Focal Adhesion** | 28 | 7.88e-07 | Actn2; Actn3; Capn2; Cav3; Col5a1; Col5a2; Col6a1; Col6a2; Col6a3; Flna; Flnc; Hgf; Itga11; Itga9; Itgb3; Itgb5; Kdr; Lama4; Lamb1; Mylpf; Pdgfrb; Pik3r5; Pip5k1c; Rac1; Rac2; Rasgrf1; Thbs2; Vcl |
| **Metabolic Pathways** | 94 | 2.85e-06 | Acaa2; Aco2; Acsl6; Ada; Akr1b8; Aldh18a1; Aldh3b1; Aldh6a1; Aldoa; Alg8; Aprt; Atp5a1; Atp5j; Atp6ap1; Atp6v0d2; B3galt2; B3gnt3; B4galnt1; B4galt1; Cd38; Chpf2; Chpt1; Chsy1; Cmbl; Coq7; Cox4i1; Cox5a; Cox6a2; Cox8b; Cyc1; Dad1; Fbp2; Fh1; Fuk; Galns; Galnt10; Gapdh; Gba; Gbe1; Gfpt1; Gmppa; Gmppb; Gns; Gusb; H2-Ke6; Hadha; Hk3; Idh3a; Inpp5a; Itpk1; Ldhb; LOC100046079; Mccc1; Mdh2; Mtap; Mthfd1l; Naglu; Ndufa2; Ndufa6; Ndufa9; Ndufb4; Ndufb8; Ndufb9; Ndufs2; Ndufs6; Ndufs7; Ndufv1; Nos1; P4ha1; Pck1; Pdhb; Pfkl; Pfkm; Pigs; Pik3c2g; Pip5k1c; Pla2g2e; Pla2g7; Plcb2; Rpn1; Sdhb; Sdhc; Sptlc2; St3gal2; St8sia5; Sucla2; Tcirg1; Tm7sf2; Tpi1; Tst; Ugp2; Uqcrc1; Uqcrc2; Uros |
| **Hypertrophic Cardiomyopathy** | 15 | 1.42e-05 | Cacna1c; Cacna1d; Cacnb1; Cacng1; Des; Itga11; Itga9; Itgb3; Itgb5; Prkaa2; Prkab2; Sgca; Sgcg; Tgfb1; Tpm4 |

Pathway analysis of differentially expressed genes between day 14 wounds and normal skin in *Mus*.
