# Supplementary material for: A Comparative Analysis of Gene Expression Profiles during Skin Regeneration in Mus and Acomys
Source: PLoS One. 2015 Nov 25;10(11):e0142931. doi: 10.1371/journal.pone.0142931 (PMC4659537; doi:10.1371/journal.pone.0142931)
Supplement: S6 Table — Pathway analysis of differentially expressed genes between day 14 wounds and normal skin in Acomys. (DOCX) [file pone.0142931.s007.docx]

**Supplemental Table 6. Pathway Analysis of *Acomys* day 14 wounds**

| **Pathway Name** | **# of Genes** | **p-value** | **Entrez Gene ID** |
| --- | --- | --- | --- |
| **Insulin Signaling Pathway** | 7 | 0.0002 | Hk2; Irs1; Map2k1; Pik3r1; Prkag2; Prkar2b; Shc3 |
| **VEGF Signaling Pathway** | 5 | 0.0004 | Map2k1; Pik3r1; Ppp3cb; Ptgs2; Vegfa |
| **Osteoclast Differentiation** | 6 | 0.0005 | Fosl2; Il1a; Map2k1; Ncf4; Pik3r1; Ppp3cb |
| **Chemokine Signaling Pathway** | 7 | 0.0009 | Ccl3; Ccl4; Jak2; Lyn; Map2k1; Pik3r1; Shc3 |
| **Focal Adhesion** | 7 | 0.0016 | Ccnd2; Itgav; Map2k1; Pik3r1; Shc3; Thbs1; Vegfa |
| **Toll-like Receptor Signaling Pathway** | 5 | 0.0017 | Ccl3; Ccl4; Lbp; Map2k1; Pik3r1 |
| **Pathways in Cancer** | 9 | 0.0018 | Ar; Bmp4; Fgfr2; Itgav; Map2k1; Pik3r1; Ptgs2; Slc2a1; Vegfa |
| **Leishmaniasis** | 4 | 0.0021 | Il1a; Jak2; Ncf4; Ptgs2 |
| **Adipocytokine Signaling Pathway** | 4 | 0.0027 | Irs1; Jak2; Prkag2; Slc2a1 |
| **Renal Cell Carcinoma** | 4 | 0.0031 | Map2k1; Pik3r1; Slc2a1; Vegfa |

Pathway analysis of differentially expressed genes between day 14 wounds and normal skin in *Acomys*.
